# Does Proprioceptive Neuromuscular Facilitation-Based Hamstring Stretching Influence Deep Cervical Flexor Muscle Endurance?

**DOI:** 10.3390/life15071019

**Published:** 2025-06-26

**Authors:** Altay Kosova, Omer Osman Pala

**Affiliations:** Department of Physiotherapy and Rehabilitation, Faculty of Health Sciences, Bolu Abant İzzet Baysal University, Bolu 14030, Turkey; fztkosova@outlook.com

**Keywords:** hamstrings, muscle stretching exercises, muscular endurance, neck, proprioceptive neuromuscular facilitation

## Abstract

**Aim:** To assess the acute effects of proprioceptive neuromuscular facilitation (PNF), stretching was applied to the hamstring muscles to evaluate deep cervical flexor endurance. Potential variables correlating with endurance adaptations were examined. **Methods:** This randomized controlled trial performed between September 2023 and June 2024 included healthy female university students aged 18–25 years. Participants were randomly assigned to either the control or the PNF group. Variables included age, height, weight, body mass index, Beighton score, hamstring flexibility, and deep cervical flexor muscle endurance; correlations between changes in hamstring flexibility and DCF endurance were explored. Hamstring flexibility was assessed using the Passive Knee Extension Test, and deep cervical flexor endurance was assessed using the Cranio-Cervical Flexion Test. The PNF group received hold–relax exercises while controls did not receive any intervention. **Results:** The study included 32 control participants and 32 individuals in the PNF group. The PNF group was marginally but significantly older than the control group [22 (21–23) vs. 21 (21–22); *p* = 0.038]. At baseline, the PNF group showed greater hamstring flexibility (*p* = 0.010). Both groups showed significant improvements in hamstring flexibility (*p* < 0.001 for both), but the improvement in the PNF recipients was far greater (*p* < 0.001). Regarding deep neck flexor endurance, no significant difference was observed between the groups at baseline (*p* = 0.958) or in final measurements (*p* = 0.244), although both groups showed significant improvements from baseline (*p* < 0.001 for both). There were no significant correlations between the change in deep neck flexor endurance and any of the examined variables. **Conclusions:** Our study found that a single session of PNF stretching significantly improved hamstring flexibility but did not immediately enhance deep neck flexor endurance. This emphasizes the need for further research into longer-term interventions to assess whether interventions on hamstring flexibility can improve cervical function.

## 1. Introduction

The Superficial Back Line (SBL) is a large fascial network extending throughout the posterior aspect of the body, from the head to the feet [1]. It comprises multiple smaller fascia structures, including the epicranial aponeurosis, occipital protuberance, erector spinae, sacrum, sacrotuberous ligament, ischial tuberosity, hamstring muscles, gastrocnemius muscles, and plantar fascia [2]. The SBL facilitates extensor movement and is crucial in maintaining postural alignment, which is bound to its flexibility and tension [1,2].

The interconnectedness of the SBL network indicates that alterations in the functioning of different components would be expected to have implications on other components [2,3]. One example is the influence of hamstring muscle tension on the cervical musculature and fascia. The hamstring muscles are located posteriorly on the thigh and include the biceps femoris, semimembranosus, and semitendinosus muscles [4]. Their flexibility and function directly impact pelvic positioning and knee joint movements [5]. Studies have also demonstrated that stretching exercises targeting the hamstring muscles can improve range of motion in the cervical joint and can correct posture [6,7]. Head and neck posture are primarily maintained by deep cervical flexor muscles, particularly the longus colli and longus capitis, which are located on the anterior surface of the cervical vertebrae [8]. Weakness or dysfunction in these muscles can necessitate overactivation of superficial muscle groups, resulting in postural abnormalities, which can lead to neck pain, cervicogenic headaches, and forward head posture [9].

Deep cervical flexor endurance is commonly assessed and trained using the Cranio-Cervical Flexion Test, which emphasizes motor control and low-load activation without recruiting superficial neck musculature. Applying proprioceptive neuromuscular facilitation (PNF) stretching techniques to the hamstring muscles may reduce tension along the SBL, thereby promoting more efficient function of the cervical musculature [6,7]. PNF techniques have also been shown to influence neuromuscular function, potentially enhancing agonist activation or reflex inhibition of antagonists, although findings are inconsistent across muscle groups. While existing literature indicates that static stretching of the hamstrings can increase cervical joint range of motion [6,10], the acute effects of PNF stretching remain unexplored in the context of deep cervical flexor endurance. The endurance of deep cervical flexor muscles is typically assessed using standard methods such as the Cranio-Cervical Flexion Test or stabilization protocols with electromyography biofeedback [11]. Given that PNF stretching enhances neuromuscular efficiency and fascial sliding through mechanisms such as autogenic inhibition and reciprocal inhibition, its application to the hamstrings may more effectively modulate tension along the SBL, thereby acutely improving the endurance capacity of cervical flexors. This hypothesis is further supported by evidence showing that PNF techniques induce broader systemic effects on postural control compared to static stretching. However, no study to date has examined whether a single session of hamstring PNF stretching can influence the endurance of deep cervical flexors, a gap that limits our understanding of the SBL’s functional connectivity in real-time movement and postural adjustments. Thus, this study aimed to investigate in healthy young women (1) the acute impact of PNF stretching of the hamstrings on deep cervical flexor endurance and (2) correlations between changes in hamstring flexibility and deep cervical flexor endurance. Given the higher likelihood of muscle- and fascia-related disorders (e.g., fibromyalgia) in women and to control for sex-related physiological variations (e.g., strength, flexibility), the study focused on female participants aged 18–25 years to ensure musculoskeletal homogeneity and minimize age-related variability [12,13,14,15].

## 2. Methods and Methods

### 2.1. Study Design and Ethical Considerations

This study was designed as a randomized controlled trial focusing on females, and its conduct adhered to the CONSORT (Consolidated Standards of Reporting Trials) guidelines. The research was carried out between September 2023 and June 2024 at the Department of Physiotherapy and Rehabilitation, Faculty of Health Sciences, Bolu Abant İzzet Baysal University, Bolu, Turkey. Study conduct followed all principles outlined in the Declaration of Helsinki and its amendments. Ethical approval was obtained from the Clinical Research Ethics Committee of Bolu Abant İzzet Baysal University (decision date: 7 February 2023, decision no.: 2023/01). Written informed consent was obtained from all participants after providing written and verbal information. The trial protocol was registered on ClinicalTrials.gov under the identifier NCT06897826.

### 2.2. Population and Participant Selection

The study population consisted of female university students aged between 18 and 25 years. The inclusion criteria were being within the specified age range and willingness to participate in the study. Exclusion criteria included hypermobility (Beighton ≥ 4), trauma, and significant flexibility asymmetry (>10°).

**Figure 1:**
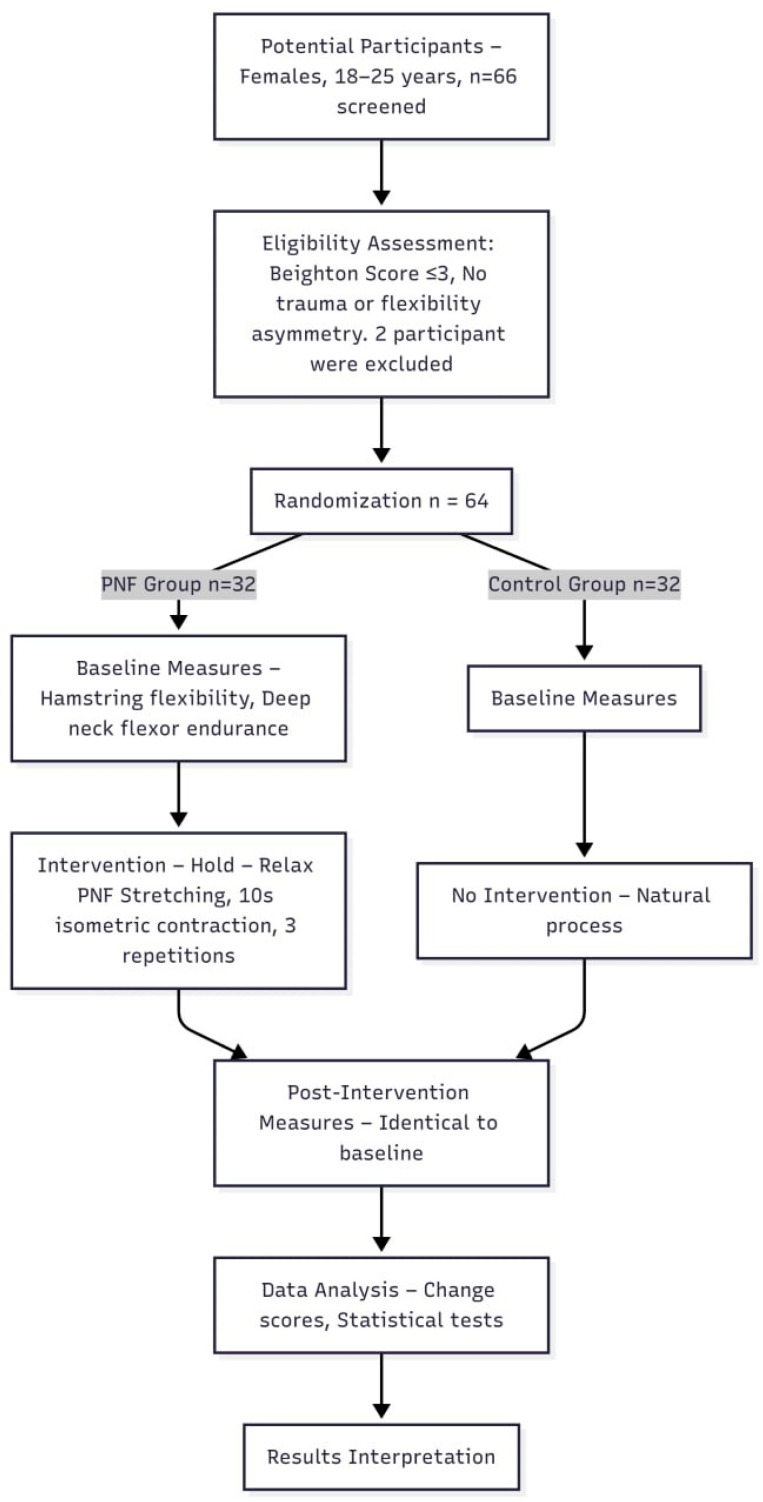
Methodology flowchart for the study. PNF: proprioceptive neuromuscular facilitation.

Randomization was performed using a computer-generated sequence (Retrieved from www.randomizer.org on 12 January 2023) by an independent researcher to assign participants into two groups: control group or PNF group undergoing hamstring stretching and control. Participants were randomly assigned to either the control group or the PNF group based on randomization performed at the time of enrollment (Figure 1).

To assess joint hypermobility, the Beighton Hypermobility Test (BHT) was used. This test is a widely accepted clinical tool for evaluating generalized ligamentous laxity [16]. The scoring system consists of five maneuvers, each contributing to a total possible score of 9 points [16,17]:

− Standing forward flexion with knees extended and palms touching the floor (1 point).
− Passive hyperextension of the fifth metacarpophalangeal joint beyond 90° (1 point per side, maximum 2 points).
− Elbow hyperextension beyond 10° (1 point per side, maximum 2 points).
− Passive opposition of the thumb to the volar aspect of the forearm (1 point per side, maximum 2 points).
− Knee hyperextension beyond 10° (1 point per side, maximum 2 points).

Participants with a total score of ≤3 were included in the study to exclude generalized hypermobility, as scores ≥ 4 are indicative of joint hypermobility syndrome.

According to descriptive statistics (effect size = 0.417) in the study by Hyong, In et al. [18], a total sample size of 63 was determined to achieve 90% power for a two-sided 0.05 significance level (alpha error). Sample size was calculated using G*Power 3.1.9.7 software [19].

### 2.3. Variables and Data Collection

The study included the following variables: age, height, weight, body mass index (BMI), BHT score, hamstring flexibility, and deep neck flexor endurance. Data were collected before and after the intervention for both groups. The primary outcome variable was deep cervical flexor endurance. Secondary outcomes included hamstring flexibility. Additionally, correlations between changes in these variables were analyzed to explore potential mechanistic relationships.

### 2.4. Assessment Methods

Hamstring flexibility was assessed using the Passive Knee Extension Test, a reliable method with an intraclass correlation coefficient of 0.88. Participants were positioned supine with their contralateral limb fully extended and stabilized, while the ipsilateral hip was flexed to 90 degrees. The examiner then passively extended the knee until reaching the point of maximal tolerable resistance in the hamstring muscles, at which point the knee angle was measured using a 30 cm plastic universal goniometer (Baseline^®^ Model 12-1000, Fabrication Enterprises Inc., White Plains, NY, USA), aligned with anatomical landmarks including the lateral femoral condyle, fibular head, and lateral malleolus [20]. Baseline measurements were recorded for all participants prior to any intervention. Following the application of the PNF technique in the experimental group, post-intervention measurements were taken using the same protocol. The control group underwent reassessment after an equivalent time period without intervention.

Deep neck flexor endurance was measured using the Cranio-Cervical Flexion Test with a biofeedback pressure sensor (Stabilizer, Chattanooga Group Inc, Hixson, TN, USA). Participants lay supine with their heads in a neutral position, and the pressure sensor was placed in the cervical curve and inflated to 20 mmHg. They were instructed to perform a gentle chin tuck without activating the sternocleidomastoid muscle. The test consisted of five progressive stages (22, 24, 26, 28, and 30 mmHg), with each stage maintained for 10 s and a 10 s rest between stages. The test was terminated if a pressure deviation of more than 2 mmHg occurred, and endurance was calculated based on the number of successfully completed stages [21,22]. Deep neck flexor endurance was measured at baseline and immediately post-intervention using identical protocols. The control group was reassessed after an equivalent time interval without intervention. Knee extension angles < 40° were considered indicative of hamstring tightness based on prior studies [23]. Normative values for the Cranio-Cervical Flexion Test in healthy individuals range from 26–30 mmHg or completing 4–5 levels.

Change scores (post-intervention minus baseline) for hamstring flexibility and deep neck flexor endurance were also calculated and analyzed separately to assess treatment effects.

### 2.5. Intervention Protocol

The PNF group underwent a hold–relax PNF stretching technique for the hamstrings. The hold–relax PNF technique was selected due to its documented efficacy in enhancing flexibility and potential reflex inhibition effects on distant muscle groups, aligning with our hypothesis. Participants lay supine while the physiotherapist passively lifted the extended leg until resistance was felt. At this position, they were instructed to perform an isometric contraction of the hamstrings at 50% of their maximum effort for 10 s, followed by relaxation and passive movement into the newly available range. This process was repeated three times. The control group did not receive any stretching intervention [21,22].

All assessments and interventions were performed by a physiotherapist in a one-on-one setting. Measurements were taken at baseline and immediately after the intervention to evaluate the acute effects of PNF stretching on hamstring flexibility and deep neck flexor endurance.

### 2.6. Statistical Analysis

All analyses were performed using IBM SPSS Statistics for Windows, Version 27.0 (IBM Corp., Armonk, NY, USA). The statistical significance value was accepted as *p* < 0.05. The Shapiro–Wilk test was used to determine whether continuous variables were normally distributed. For numerical data, descriptive statistics were presented using mean ± standard deviation for normally distributed variables while median (25th percentile–75th percentile) was used for non-normally distributed variables. Frequency and percentage were described for categorical variables. Between groups, comparisons of continuous variables were performed using the Student’s *t*-test or the Mann–Whitney U test depending on parametric assumptions. Between groups, comparisons of categorical variables were performed using the Fisher–Freeman–Halton test. Comparisons of repeated measurements were performed using the Wilcoxon signed ranks test due to non-normality of distribution. To mitigate baseline group differences, analyses were based on change scores (post–pre) for both outcomes, rather than absolute post-test values. ANCOVA was not performed due to sample size constraints. Correlation analyses were performed to explore potential relationships between changes in deep cervical flexor endurance and demographic, anthropometric, and flexibility variables using Spearman correlation coefficients due to non-normal data distribution. Effect sizes were calculated as Cohen’s d for continuous variables and Cramer’s V for categorical variables.

## 3. Results

**Figure 2:**
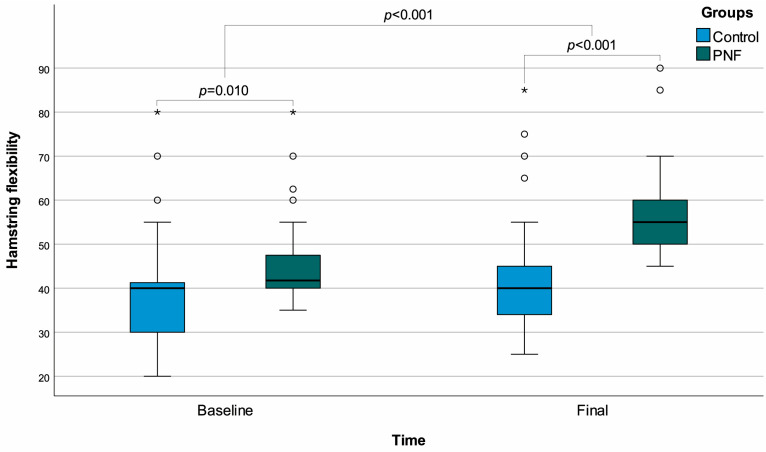
Box plots of hamstring flexibility with regard to time and groups. ◦: Outliers, *: Extreme outliers.

A total of 64 participants were included, with 32 assigned to the control group and 32 to the PNF group. No participant withdrawals were observed throughout the study duration. There was a minor but significant difference in age between the control and PNF groups, with a median age of 21 (21–22) in controls and 22 (21–23) in PNF recipients (*p* = 0.038). In terms of hamstring flexibility, the baseline values differed between the groups, with the PNF group showing a higher initial value (*p* = 0.010). In the final measurements, a significant increase was observed in the PNF group (*p* < 0.001), and both groups exhibited significant improvements (*p* < 0.001 for both) (Figure 2).

**Figure 3:**
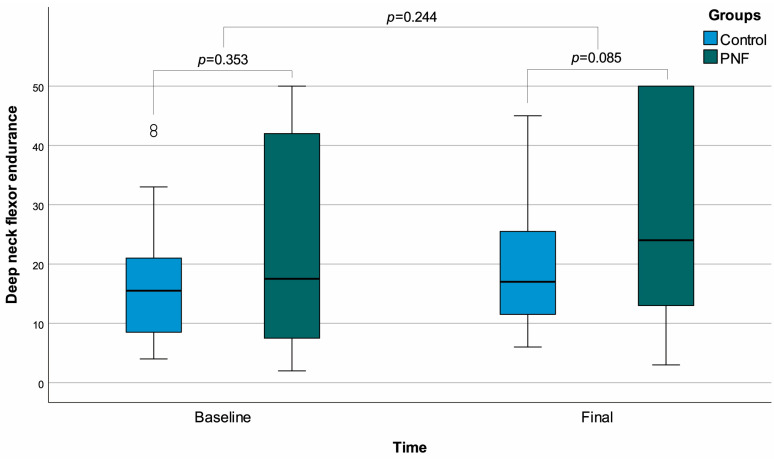
Box plots of deep neck flexor endurance with regard to time and groups. ◦: Outliers.

However, when evaluating the change in flexibility, the increase was far greater in PNF recipients compared to controls (*p* < 0.001). Regarding deep neck flexor endurance, no significant difference was found between the groups in baseline or final measurements, although both groups demonstrated significant improvements over time (*p* < 0.001 for both). The changes in endurance were also similar in the two groups (*p* = 0.244) (Table 1) (Figure 3).

There were no significant correlations between the change in deep neck flexor endurance and age, height, weight, BMI, BHT score, baseline and final hamstring flexibility, and change between final and baseline values of hamstring flexibility, regardless of group (Table 2).

## 4. Discussion

This study aimed to investigate the acute effects of PNF stretching applied to the hamstring muscles on deep neck flexor endurance in healthy individuals. Our results demonstrated that PNF stretching of hamstrings produced no differential effects on neck flexor endurance between groups. While the stretching intervention significantly improved hamstring flexibility compared to controls, these changes were not associated with neck endurance outcomes. Notably, changes in deep neck flexor endurance showed no significant correlations with age, anthropometric measures (height, weight, BMI), BHT score, or hamstring flexibility parameters (baseline, post-intervention, or change scores).

Hamstring flexibility is crucial for posture and contributes to musculoskeletal function. The loss of flexibility has been associated with lumbar and cervical impairments [24]. PNF stretching improves muscle elasticity and is a widely used and efficacious technique for such purposes [7,16]. The considerable increase in flexibility observed among PNF recipients supports the efficacy of this technique. Although baseline hamstring flexibility showed a significantly higher value in the PNF group, the between-group difference in flexibility improvement remained significant after accounting for baseline values. Previous studies have similarly reported that PNF stretching enhances hamstring flexibility more effectively than static stretching [7,25]. Moreover, interventions targeting other regions of the SBL, such as plantar fascia release, have also been shown to improve hamstring flexibility [26]. These findings align with the concept of myofascial continuity, where mechanical tension in one part of the kinetic chain can influence distant regions [27]. Thus, our results contribute to the growing evidence supporting the effectiveness of PNF stretching in increasing hamstring flexibility.

The absence of significant correlations between hamstring flexibility changes and deep cervical flexor endurance adaptations challenges the theoretical framework of immediate myofascial chain interactions. Despite substantial improvements in hamstring flexibility following PNF stretching, these local changes did not translate to measurable endurance gains in the distant cervical musculature, suggesting that acute mechanical effects along the Superficial Back Line may not extend to functional muscle performance parameters.

Deep neck flexor endurance is critical for cervical spine stability and postural control, particularly in individuals with neck pain [6]. While interventions improving flexibility in one muscle group have been associated with positive impacts on distant muscles via myofascial connections [6,7,28], our study did not find a significant between-group difference in deep neck flexor endurance following hamstring PNF stretching. This lack of relationship challenges the mentioned studies in terms of the proposed impacts on distant muscles, at least in terms of muscular endurance metrics. Both groups showed significant within-group improvements over time, likely due to a learning effect or repeated testing, which might also have been influenced by the fact that our subjects were young adults without clinical complaints. This trend may reflect a learning effect, as both groups underwent repeated testing within a short time frame. Familiarity with the assessment tool or increased task-specific awareness may have contributed to apparent gains.

Various studies on the SBL have clearly demonstrated the connections between the muscles in this group [6,10,29,30,31]. In a cross-sectional study examining the myofascial relationship between hamstring flexibility and cervical function in healthy young adults, researchers found that participants with limited hamstring flexibility demonstrated significantly lower deep neck flexor endurance compared to those with normal flexibility, though no differences were observed in cervical range of motion [6]. In a randomized controlled trial, the effectiveness of combining suboccipital muscle inhibition (SMI) with hamstring hold–relax agonist contraction (HR-AC) was investigated in 34 patients with neck pain and hamstring tightness. The study found that the SMI+HR-AC treatment, administered three times per week for two weeks, yielded significantly better results in pain, cranio-vertebral angle, and neck disability index compared to the group that received SMI alone [31]. Stretching exercise appears to be crucial for obtaining measurable outcomes. For instance, a randomized controlled trial on patients with neck pain that evaluated the immediate effects of static stretching and PNF stretching on hamstring tightness and cervical parameters described no between-group differences with respect to straight leg raise (SLR), cranio-vertebral angle (CVA), or cervical range of motion (CROM). However, both stretching methods significantly improved SLR, CVA, and CROM within each group after a single session [10]. In a cross-sectional study investigating the relationship between forward head posture (FHP) and lower extremity muscle tightness, researchers found a weak but significant correlation (r = 0.300, *p* = 0.034) between CVA and right hamstring tightness in normal-BMI males. However, no strong associations were observed between FHP and calf muscle tightness or in other demographic groups [29]. While many studies report associations between remote muscle interventions and cervical outcomes, the heterogeneity in protocols and outcomes—as well as small effect sizes—highlight the need for cautious interpretation of such findings.

Despite weak effect sizes being reported, emerging evidence from systematic reviews supports the existence of myofascial chains that underly biomechanical continuity throughout the SBL (plantar fascia, gastrocnemius, hamstrings, and spinal erector muscles). These interactions may explain clinical associations between hamstring tightness and cervical dysfunction [30]. A randomized study found that both static stretching and remote myofascial release applied to either the plantar fascia or cervical region significantly improved hamstring elasticity, with combined interventions showing additive benefits [32]. Complementary findings supporting this unidirectional relationship also exist, and have revealed that suboccipital muscle inhibition techniques and cranio-cervical flexion exercises increase hamstring flexibility and improve postural metrics (popliteal angle, CVA) [33]. Furthermore, instrument-assisted soft tissue mobilization applied to proximal or distal SBL components were found to increase hamstring flexibility and overall functioning [34]. However, our results appear to suggest that these effects might not be bidirectional, casting doubt on whether hamstring exercises can indeed improve cervical muscle characteristics.

The lack of a significant between-group difference in the current study may be attributed to several clinically relevant factors. First, the acute nature of the post-interventional measurement may have prevented measurable changes in deep neck flexor endurance. In relation, adaptations in muscle or fascia would require longer-term stretching protocols, possibly involving neural adaptations or structural changes in the muscle–tendon unit. Therefore, it may be advisable to perform longer-term studies with sustained or intensive interventions to assess endurance outcomes. Second, the presence of baseline differences in age and hamstring flexibility between groups may have confounded the results; however, we also examined scores to assess differences from baseline, which would be expected to mitigate this limitation. Third, while the SBL theory suggests mechanical interactions between hamstring and cervical muscles [6], the acute neuromuscular effects of PNF stretching may not translate to endurance improvements in distant muscle groups. This dissociation between flexibility and endurance responses has important implications for clinical practice, suggesting that comprehensive rehabilitation programs should address these qualities separately.

Our findings contribute to the growing body of literature investigating the functional implications of myofascial connectivity along the SBL. Although various studies have demonstrated associations between hamstring flexibility and cervical function, particularly in chronic interventions or combined treatment protocols [6,10,29,30,31], the present results suggest that acute hamstring stretching alone may not acutely improve deep neck flexor endurance. Nevertheless, it might be beneficial to consider the possibility of delayed or cumulative effects over time, especially when combining local and distant interventions. Future research should explore longer-duration protocols and utilize imaging studies to assess myofascial adaptations.

Despite showing the strong impact of PNF stretching on hamstring flexibility, the baseline differences between the groups should be considered as limitations. The PNF recipients were marginally older and initially had higher hamstring flexibility. Although we accounted for these differences by analyzing the amount of change rather than absolute values, the results may still be influenced by underlying differences. Second, the deep neck flexor endurance test may not solely assess muscular endurance, as it also involves cognitive factors such as attention and motor learning, potentially introducing variability. This is supported by the observed learning effect, with both groups showing significant improvements over time. Third, the single-session design limits the generalizability of our findings to chronic adaptations, and future studies should investigate the long-term effects of PNF stretching on both hamstring flexibility and deep neck flexor endurance. Additionally, the present results are only relevant for females and we did not consider menstrual cycle-related variations, which could influence muscle flexibility and endurance. The lack of other stretching modalities that may lead to different effects on deep neck flexor endurance (eg, neurodynamic techniques) is another factor to consider before drawing conclusions from this study. Therefore, future studies comparing various stretching approaches are warranted to clarify whether the type of intervention plays a role in the observed neuromuscular responses. This study did not include neurodynamic assessments (e.g., Slump test) to evaluate the potential influence of neural tension on flexibility measurements. Finally, the current literature lacks sufficient studies that bidirectionally examine the relationship between hamstring flexibility and deep neck flexor endurance, particularly in the context of acute interventions.

## 5. Conclusions

Our study demonstrated that a single session of PNF stretching significantly improves hamstring flexibility but does not affect deep neck flexor endurance in young adult females without any neuromuscular complaints. Deep neck flexor endurance changes did not correlate with age, anthropometrics, BHT score, or hamstring flexibility measures. These findings suggest that (1) PNF effectively targets local flexibility but may not immediately influence distant muscles through myofascial connections, and (2) rehabilitation programs requiring cervical endurance improvements should incorporate targeted interventions rather than relying solely on remote stretching approaches. However, while biomechanical and anatomical studies suggest fascial continuity, the neuromuscular translation of such connectivity—especially in terms of endurance metrics—remains theoretical and likely influenced by multiple intervening factors such as neural adaptation, task learning, and individual variability. Clinicians should consider these results when designing rehabilitation programs, with the understanding that remote stretching interventions may not produce positive outcomes for the neck muscles. Future research should prioritize longer-term and repeated interventions, explore different stretching modalities, and incorporate neurodynamic assessments to clarify the bidirectional influence between lower and upper body myofascial chains.

## Figures and Tables

**Table 1:** Summary of demographics and measurements with regard to groups.

	Groups			
	Control (*n* = 32)	PNF (*n* = 32)	Test Statistic/ *p* (Between Groups)	Effect Size
Age	21 (21–22)	22 (21–23)	**U = 363.5/*p* = 0.038 ^‡^**	Cohen’s d = 0.537
Sex, female	32 (100.00%)	32 (100.00%)	N/A	N/A
Height, m	1.64 ± 0.07	1.65 ± 0.05	t = −0.385/*p* = 0.702 ^†^	Cohen’s d = 0.096
Weight, kg	59 (51.5–66)	62.5 (57–72)	U = 399/*p* = 0.129 ^‡^	Cohen’s d = 0.350
Body mass index, kg/m^2^	22.30 ± 2.95	23.78 ± 4.53	t = −1.547/*p* = 0.128 ^†^	Cohen’s d = 0.387
Beighton Hypermobility Score				
Score 0	20 (62.50%)	24 (75.00%)	Exact = 3.698/*p* = 0.328 ^¶^	Cramer’s V = 0.246
Score 1	6 (18.75%)	2 (6.25%)		
Score 2	5 (15.63%)	3 (9.38%)		
Score 3	1 (3.13%)	3 (9.38%)		
Hamstring flexibility				
Baseline	40 (30–41.25)	41.75 (40–47.5)	**U = 322.5/*p* = 0.010 ^‡^**	Cohen’s d = 0.397
Final	40 (34–45)	55 (50–60)	**U = 173/*p* < 0.001 ^‡^**	Cohen’s d = 1.097
Test statistic *p* (within groups)	**z = −3.905** ***p* < 0.001 ^§^**	**z = −5.068** ***p* < 0.001 ^§^**		
Effect size	Cohen’s d = 1.061	Cohen’s d = 3.458		
Change ^(a)^	2.5 (0–5)	10 (10–14.5)	**U = 9/*p* < 0.001 ^‡^**	Cohen’s d = 3.310
Deep neck flexor endurance				
Baseline	15.5 (8.5–21)	17.5 (7.5–42)	U = 443/*p* = 0.353 ^‡^	Cohen’s d = 0.448
Final	17 (11.5–25.5)	24 (13–50)	U = 384/*p* = 0.085 ^‡^	Cohen’s d = 0.592
Test statistic *p* (within groups)	**z = −4.478** ***p* < 0.001 ^§^**	**z = −4.292** ***p* < 0.001 ^§^**		
Effect size	Cohen’s d = 1.352	Cohen’s d = 0.940		
Change ^(a)^	2 (1–4)	3.5 (0.5–6.5)	U = 426/*p* = 0.244 ^‡^	Cohen’s d = 0.546

Descriptive statistics are presented using mean ± standard deviation for normally distributed continuous variables, median (25th percentile–75th percentile) for non-normally distributed continuous variables and frequency (percentage) for categorical variables. ^(a)^ Difference between final and baseline measurements, positive values represent increase. ^†^ Student’s *t*-test, ^‡^ Mann–Whitney U test, ^¶^ Fisher–Freeman–Halton test, ^§^ Wilcoxon signed ranks test. Statistically significant *p* values are shown in bold. N/A: not applicable. Bold expressions indicate statistically significant variables.

**Table 2:** Correlations between change in deep neck flexor endurance and other variables.

	All Individuals (*n* = 64)		Control Group (*n* = 32)		PNF Group (*n* = 32)	
	r	*p*	r	*p*	r	*p*
Age	−0.045	0.724	−0.206	0.259	−0.036	0.844
Height, m	0.089	0.484	−0.148	0.419	0.234	0.196
Weight, kg	0.093	0.465	0.128	0.486	0.064	0.729
Body mass index, kg/m^2^	0.054	0.669	0.211	0.247	−0.066	0.721
Beighton Hypermobility Score	−0.115	0.365	−0.031	0.868	−0.156	0.392
Hamstring flexibility, baseline	−0.072	0.571	−0.077	0.677	−0.176	0.335
Hamstring flexibility, final	−0.023	0.857	−0.145	0.427	−0.157	0.391
Hamstring flexibility, change	0.054	0.670	−0.301	0.094	−0.061	0.742

r: Spearman correlation coefficient.

## Data Availability

The data presented in this study are available on request from the corresponding author.
